# An Analysis of the Policy Environment Surrounding Noncommunicable Diseases Risk Factor Surveillance in Kenya

**DOI:** 10.3934/publichealth.2014.4.256

**Published:** 2014-12-02

**Authors:** Rosemary Mamka Anyona, Maximilian de Courten

**Affiliations:** 1 School of Global Health, University of Copenhagen, Copenhagen, Denmark; 2 Centre for Chronic Disease, College of Health & Biomedicine, Victoria University, Melbourne, Australia

**Keywords:** Kenya, policy, noncommunicable diseases, chronic diseases, risk factors, NCD surveillance, risk factor surveillance

## Abstract

Kenya is a developing country in sub-Saharan Africa, facing a triple disease burden, with an increase in non-communicable diseases (NCDs); uncontained infectious diseases; coupled with significant morbidity and mortality from environmental causes such as droughts and flooding. The limelight has been on infectious diseases, leaving few resources for NCDs. As NCDs start to gain attention, it is becoming apparent that essential information on their epidemiology and risk factor trends—key in evidence-based decision-making—is lacking. As a consequence, policies have long relied on information derived from unreliable data sources such as vital registries and facility-level data, and unrepresentative data from small-scale clinical and academic research. This study analyzed the health policy aspects of NCD risk factor surveillance in Kenya, describing barriers to the successful design and implementation of an NCD risk factor surveillance system, and suggests a strategy best suited for the Kenyan situation. A review of policy documents and publications was augmented by a field-study consisting of interviews of key informants identified as stakeholders. Findings were analyzed using the Walt and Gilson policy analysis triangle. Findings attest that no population baseline NCD burden or risk factor data was available, with a failed WHO STEPs survey in 2005, to be undertaken in 2013. Despite the continued mention of NCD surveillance and the highlighting of its importance in various policy documents, a related strategy is yet to be established. Hurdles ranged from a lack of political attention for NCDs and competing public interests, to the lack of an evidence-based decision making culture and the impact of aid dependency of health programs. Progress in recognition of NCDs was noted and the international community and civil society's contribution to these achievements documented. While a positive outlook on the future of NCD surveillance were encountered, it is noteworthy that overcoming policy and structural hurdles for continued success is imperative.

## 1. Introduction

Global changes in demographic, social, economic, ecological and biological factors have caused a shift in disease profiles, with the long-standing predominance of infectious diseases subsiding, giving way to an emergence of noncommunicable diseases (NCDs) [1],[2]. There are 63% of the 57 million global deaths that occurred in 2008 were due to NCDs, 80% of these occurring in low and middle income countries [3]. The unique identifier of NCDs is their common risk factor pattern, which include a modifiable behavioral component and physiological component. The modifiable risk factors, if mitigated through easily implementable and cost-effective methods, can lead to significant reduction in NCD mortality [3]. Through these measures, the UN and WHO target to reduce premature NCD mortality by 25% by the year 2025.

Kenya, a developing sub-Saharan African country, is facing a triple disease burden [4], with an increase in NCDs overlapping an uncontained burden of infectious diseases, coupled with significant morbidity and mortality from environmental causes such as droughts and flooding. According to the Ministry of Health Annual Status Report 2007, the most recently available data from the government, NCDs contribute over half of the top 20 causes of morbidity and mortality, with mortality rising from 31.8% in 2002 to 33% in 2007 [5]. Separately, the WHO Kenya country profile estimates that NCDs contribute 28% of all-cause mortality in Kenya annually [6]. In the absence of accurate data, conflicting figures are often quoted as hereby demonstrated.

It is worth noting that Kenya's ageing population is projected to increase, with the > 50 years population increasing from 12.9% in 2009, to 17% in 2015 [5]. Likewise, hospital mortality rates are expected to rise from 50% to 60% by 2030 [7]. What's worse is that the birth rate and child and maternal mortality are not projected to reduce enough within that time frame to free up resources for focus on NCDs [3].

Action to curb the pending crisis which threatens to cripple the already fragile health system in Kenya is imperative. However, this action requires as a precursor, knowledge of the extent of the problem. Accurate data on the disease burden, risk factor profile and mortality and morbidity statistics from Kenya is scanty. Emphasis has for a long time been on infectious diseases; with underreporting, missed diagnoses, misdiagnosis, and misclassification of NCDs. Therefore, the actual extent of NCDs and their associated risk factors at population level remain largely unknown [8].

In recognition of a prevailing problem of a lack of accurate data and information on NCD mortality statistics and risk factor profiles from many countries, the global status report on NCDs 2012 urges the improvement of national-level surveillance and monitoring as a high priority agenda in the fight against NCDs, stressing that simple and sustainable systems can viably produce valuable data even in resource-poor settings [3].

Surveillance, defined as the “systematic collection and use of epidemiologic information for the planning, implementation, and assessment of disease control”, [9] was applied to NCDs during the 1968's twenty first World Health Assembly, as an expansion on existing disease surveillance, which then mainly centered on infectious diseases [9]. Three essential components of NCD surveillance include monitoring of risk factors, monitoring of morbidity and disease-specific mortality and monitoring of health system responses i.e. policies, infrastructure and human resources [3].

NCD risk factor surveillance, the first component of NCD surveillance, aims to provide a platform for systematic data collection on trends and emerging patterns of NCD behavioral and biological risk factors [10]. This information is fundamental, not only in policy and targeted intervention strategy design, but also for continuous monitoring and evaluation of the impact and efficacy of existing interventions and for predicting future trends. It also helps create models of socio-behavioral health determinants and define the impact of factors beyond strategic interventions, such as the effects of the political environment, on their patterns.

By the time of the start of this research (April 2013), no information was available on any attempt to undertake a survey on NCD risk factors in Kenya, nor on the existence of an NCD risk factor surveillance initiative or strategy. Kenyan policy makers alluded to the importance of a surveillance system in the country, most prominently at the First National Forum on Noncommunicable Diseases in 2011 [11], where the development of a surveillance strategy, amongst other calls to action for NCDs, was established as a conclusive agenda. The bottleneck where the policy delineating the implementation of NCD surveillance is impeded remained unclear.

Pursuant to this gap in information, this study was undertaken analyze the policy environment surrounding NCD risk factor surveillance in Kenya, attempting to identify and describe hurdles, if any, which have been barriers to the successful design and implementation of an NCD risk factor surveillance strategy.

## 2. Materials and Method

### 2.1. Study design

The study was a prescriptive analysis of policy case study of the Kenyan policy environment surrounding noncommunicable disease risk factor surveillance. Public health surveillance is a matter of public policy, and therefore, in attempting to understand the situation, a study of the policy environment surrounding NCDs was found to be the most tenable approach pursuant to this aim.

Health policy, in this case “is assumed to embrace courses of action (and inaction) that affect the set of institutions, organizations, services and funding arrangements of the health system” [12].

The study was based on a policy analysis literature through review of the augmented by qualitative data collection amongst stakeholders.

**Figure 1.**
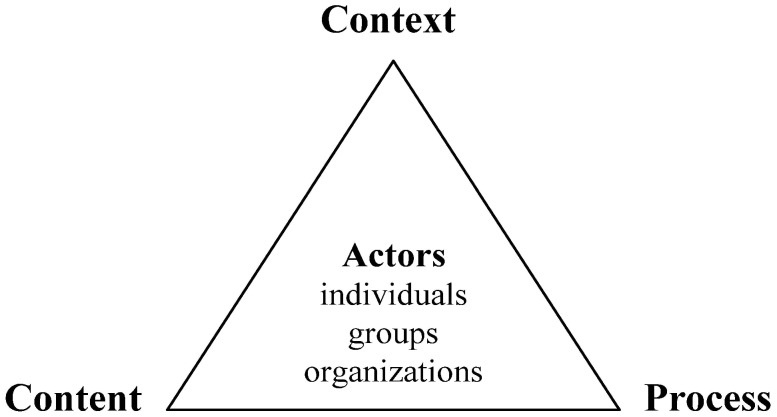
The Walt and Gils on policy triangle.

The study was designed based on the Walt and Gilson policy triangle [13], therefore, relevant data was collected according to the four key policy areas. The framework breaks down policy analysis into process, context, actors and policy content (Figure 1).

Purposeful sampling was used to identify the relevant key informants. This was done by performing a thorough stakeholder analysis. Relevant organizations and institutions were identified, followed by the identification of the relevant offices within the institutions. The office holders were deemed to have influence, direct or indirect, to the policy process involved in NCD surveillance.

Key informants in positions relevant to NCD risk factor surveillance were identified for interviewing from each of these groups.

Snowball sampling was also used during the data collection process, with key informants explicating other offices, organizations or persons from whom relevant information could be attained.

### 2.2. Data collection tools

The field study entailed data collection through interviews guided by open ended, researcher administered questionnaires. The questionnaires were formulated based on the objectives, and were intended as a guide for obtaining the necessary information from the key informants.

A thorough literature search was carried out to identify material for analysis. An initial search for information from official web pages representing the stakeholders was carried out, which aided in identification and at times access to policy documents, reports, releases and publications. Further documents not accessible online were requested for from the relevant offices.

Further, keyword and mesh terms searches such as “noncommunicable disease risk factor surveillance”, “disease surveillance”, “NCD surveillance policies”, “health policy”, “policy analysis” were carried out on internet search engines, including Google and Google Scholar and on health research databases including PubMed, Elsevier, Science Direct and Web of Science. Press releases, newspaper articles and other unpublished grey literature were also used as necessitated.

Data triangulation was achieved by augmenting the analysis of available literature and policy documents with stakeholder interviews, and theory triangulation done by application of various theoretical frameworks in the analysis of findings; to ensure validity of the research.

### 2.3. Fieldwork and data collection

- The principal investigator conducted the field study between the dates of 20th April 2013 and 20th May 2013. Questionnaire guided interviews were conducted amongst the following key informants:
- Head of Non-Communicable Diseases Division, MoH, Kenya
- Non-Communicable Diseases Advisor to World Health Organization Country Office, Kenya
- The immediate former Head of Non-Communicable Diseases Division, MoH, Kenya
- Research Officer, Kenya Medical Research Institute, Kenya
- Chair of Kenya Non-Communicable Disease Alliance and Chair of Kenya Cardiac Society, Kenya
- Chairman, Diabetes Management & Information Centre, Nairobi, Kenya
- Team Leader, Surveillance. Surveillance and Population-based Prevention Unit Prevention of Noncommunicable Diseases Department. World Health Organization Headquarters, Geneva

The interviews were recorded with a recording device with consent from the interviewees, who were assured of anonymity in the presentation of their responses and the highest reasonably attainable standard of discretion and confidentiality in the handling of information obtained and in the presentation of research findings.

The data collected above was transcribed verbatim, and running themes were identified. As stated above, the Walt and Gilson Policy Triangle framework of policy analysis [13] was used to deconstruct the themes and reconstruct an analytical prose which would concisely explain the policy aspects as guided by the objectives.

### 2.4. Ethical consideration

Authority to conduct the study was obtained in writing from the Ministry of Health as granted by the office of the Director of Medical Services.

Informed consent was also obtained verbally from each key informant after a thorough elaboration of the research background, in accordance with the provisions of the Declaration of Helsinki [14]. Informants were assured of the utmost level of discretion and confidentiality reasonably attainable in the handling and presentation of collected information.

## 3. Results

The findings of this study confirmed that there were currently no comprehensive baseline data on NCD indicators such as incidence, morbidity and mortality and of risk factor patterns, based on any large scale survey or that is nationally representative.

The Ministry of Health in Kenya has a Non-Communicable Diseases Division, which is staffed and currently has a budgetary allocation. There is no comprehensive NCD policy document; however, there are several disease specific strategies and guidelines, namely: Kenya Cancer Control Strategy, Diabetes Control Strategy and Treatment Guidelines and the Tobacco Control act. Other strategies are in the process of formulation, including an NCD action plan document, the national Non-Communicable Diseases Strategic Plan 2012–2017.

A national NCD risk factor survey, utilizing the WHO STEPwise approach, was carried out in 2005, but the results of the survey were deemed inadmissible by the stakeholders and policy makers, and were therefore not published and have not been used to inform any decision making. The confirmation of a failed attempt at a survey was resounding throughout all the interviews, however, no records of this were available, therefore, the explanation behind the causes of failure varied from respondent to respondent, with some recurring themes.

A repeat survey was proposed in 2009 and, according to information gleaned from the Kenya NCD Alliance office, the survey is tentatively set to be rolled out within the month of October 2014. This nation-wide household survey also utilizing the WHO STEPwise approach is being carried out by the Ministry of Health (MoH) in conjunction with the CDC, WHO and the Kenya Medical Research Institute (KEMRI).

The analysis of the results was done in accordance to the Walt and Gilson Policy Triangle [13]. The findings are therefore presented comprehensively below, utilizing the components of the triangle, Actors, Context, Process and Content.

### 3.1. Actors

The term “actors” denotes individuals and organizations (including the State) whose actions influence policy [12]. In this case, the actors were identified prior to and during data collection, and also while analyzing the findings and categorized using a top-down model; from Global, to Regional, to National and lastly Local stakeholders (Table 1) below,

**Table 1.** Classification of the stakeholders (actors).

Global stakeholders	Regional stakeholders	National stakeholders	
		State actors	Non-state actors
World Health Organization	African Union	Executive, Government of Kenya	Civil Society-Kenya Noncommunicable Diseases Alliance
United Nations	WHO,Africa Region	Ministry of Health, Government of Kenya	World Health Organization,Kenya Country Office
Centre for Disease Prevention and Control		Noncommunicable Diseases Division, MoH	Private Hospitals
Global Non-Communicable Disease Alliance		Kenya Medical Research Institute	Private Universities
		National Authority for Campaign against Alcohol and Drug Abuse (NACADA)	
		Public Universities	

### 3.2. Context

This component refers to systemic factors—political, economic and social, both national and international—which affect health policy, and will be categorized according to a system proposed by Leichter [15]. The Leichter system breaks down context into Structural Factors, Situational Factors, Cultural Factors and International Factors. Due to the significant nature of the international factors encountered, they have been discussed as situational factors.

#### 3.2.1. Structural factors

##### The current status of the health surveillance in Kenya

A disease surveillance system exists; however, it is limited to targeting diseases of “public health importance” and diseases for eradication and epidemic prone diseases, with no NCD coverage. The most successful of these is the HIV surveillance, which is done through continuous antenatal clinic data collection, augmented by five-yearly Kenya Demographics and Health Survey (KDHS), done in 1993, 1998, 2003 and 2008 [16]. KDHS is based on a nationally representative sample [17], providing estimates for rural and urban areas of the country and for each of the eight provinces (now dissolved into 47 counties). This has also included sentinel surveillance for outbreaks of infectious diseases such as poliomyelitis.

A Division of Health Information System responsible for collection, collation and analysis of data from health facilities, research institutions, census and surveys exists in MoH. Under this division, a District Health Information Software (DHIS2), a web-based software, was created to electronically manage aggregated countrywide data [18]. Despite the capturing of large amounts of data, the system suffers from problems such as lack of trained manpower and technical know-how, poor ICT infrastructure at low-level facilities (with up to 70% of patient data collected manually), lack of storage space for record-keeping and questionable quality of data which leads to non-use of the data.

Cancer registries reportedly exist in regions and large health facilities, and are hence unreliable as they are not representative. A diabetes registry was piloted but is yet to be rolled out nationally.

##### Political recognition of NCDs

The Kenyan healthcare system is grossly underfunded and unprioritized by the government and policy makers, a factor recounted by the key informants across the board as an umbrella problem overlying all that ails the system. The system, heavily funded privately through out-of-pocket payments and by donors, is decrepit and unsustainable. According to the National Health Accounts 2009/10, the total health expenditure (THE) per capita was $42, with the government contributing only 29% of the THE [19]. The government spending was 4.6% of the GDP, less than a third of the agreed upon 15% of the Abuja Declaration, of which Kenya is a signatory [20].

Furthermore, within this dilapidated system, focus has long been on infectious diseases such as malaria and HIV, and maternal and child health. In the generally underfunded system, these competing interests have taken precedence, draining the meager resources and therefore playing a major role in the lack of NCD recognition.

In 1998, the MoH responded to external pressure to form a Division of Non-Communicable Diseases (DNCD), however, as reported by key informants, the division was understaffed and underfunded, remaining dormant for almost a decade to follow. As recently as 2007, NCDs did not feature in the Ministerial annual operating plan (AOP), meaning that the division remained without a budget line, making it impossible to undertake any public NCD intervention activities. This was attributed to the lack of evidence of NCD burden (i.e. lack of data) and therefore lack of evidence of NCD importance.

However, through collaborative efforts between the division and the civil society, on whose funding, existing structure and strong support systems the division leveraged itself, the NCD agenda was further driven, finally gaining recognition and resource allocation in the form of inclusion in the 2008/9 Ministerial AOP. Another important milestone for NCDs in Kenya is the upcoming Health Sector Strategic Plan III, where three out of the six key agendas have been dedicated to NCDs [21].

Beyond the aforementioned factors, other political factors which hindered the efforts to create a policy included the fragmentation of the healthcare system. In 2008, following a highly contested presidential election, the ministry of health was fragmented into two ministries, the Ministry of Medical Services, responsible for treatment and secondary care and the Ministry of Public Health and Sanitation responsible for prevention and primary care; to accommodate the two opposing political parties which formed a coalition government. This stretched policy making efforts between the two ministries, complicating consensus. Fortunately, as of 2013, with the formation of a new government, the two will be reconsolidated.

A final and crucial factor which was also cited as hindering resource allocation to NCDs was the non-immediate visualization of results of interventions for NCDs, hence presenting little political appeal. Interventions with quickly visible results, such as those for infectious diseases, are apparently preferable, as they appeal to the public and cast the politician behind the initiative in good light. It takes years, even decades, for the results of NCD prevention and control measures to produce tangible results.

##### Civil society and the NCD agenda

A major theme in the NCD advocacy in Kenya has been the central role played by Professional and Civil society groups and research organizations in the setting of the NCD agenda in Kenya. It is noteworthy, however, that the most visible and active group was the professional associations. The organizations have been fundamental in driving the creation of, and thereafter advising and supporting the DNCD and in the lobbying for the various legislations and disease specific strategies and management guidelines. This has been accomplished through their involvement in technical working groups which coordinate initiatives for the various activities in NCD advocacy, with the DNCD leveraging itself on their financial and technical resources.

The professional societies had internal factors which hindered reaching of a collective agenda. One such factor was reported as scattered initiatives by the different disease specific societies, described as “a traditional approach where we split NCDs into different components because we came from different specialist societies. We had activities for diabetes devoid of CVD though the two diseases are actually one we ended up with a Cancer Act, respiratory initiatives leading to an Asthma Action Plan and Asthma Guidelines; a Diabetes Action Plan, something on epilepsy instead of a comprehensive NCD plan which would address common causative root of NCDs.”

The strong civil movement's impact in setting the NCD agenda in Kenya is a scenario mirrored globally. Highly visible and vocal non-state actors such as the global NCD Alliance [22], which is a the unification of four key international non-governmental organizations; the International Diabetes Federation, World Heart Federation, International Union against Tuberculosis and Lung Diseases and the Union for International Cancer Control; along with an analogous informal collaboration of academics, practitioners, and civil society organizations, the Lancet NCD Action Group, have worked hand-in-hand in the interests of promoting a unified political message and a common voice to global and national policy makers on NCDs. As an example of their influence, in the lead up to the UNHLM 2011, these groups produced comprehensive documents proposing priority actions for NCDs and proposing contents of the resolution [23].

Civil society has historically been considered weak and fragmented [24], but in the case of the Kenyan response to NCDs, despite the obvious fragmentation encountered due to the different disease entities and cause-definitions pursued by the different civil, professional and patient groups, their attempt at amalgamation of exertion in advocacy, however challenging, has been a key driving force in NCDs. Furthermore, in resource-poor countries, civil society has been a strong partner in supplementing government activities, a finding in keeping with the Kenyan NCD context. It is therefore imperative to maintain and strengthen the relationship between the state organs responsible for NCDs, and the civil society.

While civil society participation is largely encouraged, there should be an awareness of pitfalls such as “development ventriloquism”, a phenomenon where civil society experts base research and advocacy on their own objectives and from their point of view, overlooking the needs of the society in which they participate [24].

To incorporate all stakeholders and minimize shortcomings presented by their different limitations, the WHO proposes the establishment of an independent autonomous legally mandated body overarching all national activities on NCDs, with multi-sectoral composition, monitoring the MoH's NCD activities, monitoring progress on indicators (by use of surveillance risk factor and morbidity and mortality trend data) and monitoring leadership commitment. The creation of such a body would be highly advantageous, not only in ensuring progress in NCD control, but also in the implementation of a surveillance system, as one would be essential in aiding the unit's monitoring activities. No plans, however, are underway for the development of such a body, with no mention of such in the Action Plan document or by the respondents.

#### 3.2.2. Situational factors

Situational factors, variously referred to as “Focusing Events”, are described as the more or less transient, impermanent, or idiosyncratic conditions which can have an impact on policy [12].

##### International initiatives

The most consistent factor encountered in this theme was international focus on NCDs, which has gained momentum in the recent past, consequently driving the agenda locally. As recounted by informants, the build-up towards the UNHLM was a key driving force, denoting the period during which most progress was made legislatively and in strategy formulation towards NCDs.

With pressure from the WHO and support from international NCD-related bodies such as the Global NCD Alliance, the ICDRC, the World Diabetes Foundation, the Centre for Disease Prevention and Control, and others, the agenda for NCDs was set in Kenya, gaining the them recognition and focus. As an example, legislations such as the Tobacco Control Act received a lot of support from the Framework Convention on Tobacco Control (FCTC), International Development Research Centre (IDRC) and the Bloomberg Initiative.

The recently unveiled Global Monitoring Framework [25] was quoted as being a key driver and guiding document on monitoring and surveillance of NCDs and their risk factors, and their integration into the Health Management and Information System.

It was a matter of surprise and concern, according to the interviewees, that Kenya remains far behind its regional counterparts as far as surveillance is concerned, despite having a relative competitive advantage as far as resources are concerned.

##### Media

The second recurring theme in situational factors is the media, which has given NCDs and their lack of political recognition great attention in the recent past. Media has incorporated NCDs into their health segments with focus on behaviour change, nutrition and providing information on diseases such as cancer and diabetes. This attention has been highly beneficial in national sensitization and most importantly, has acted as a platform for the civil society and professional bodies on which to relay their message and lobby for political recognition and resource allocation.

##### Cancer diagnosis amongst key political figures

In 2011, the two health ministers in Kenya were both diagnosed with cancer. Their public struggle with the disease was a crucial driving force for NCD agenda. Then Minister of Medical Services detailed his struggle with prostate cancer in newspaper articles. *East African Standard* [26], a widely circulated news publication ran articles detailing his struggle and its effect on his renewed single-mindedness about fighting cancer in Kenya as follows: “Sitting at the apex of Kenya's health services, and having come face to face with the cruelty of cancer, he is worried about the chances of survival for those not as privileged as himself, who could afford long treatment abroad”.

The Minister expressed his dismay at the status quo of cancer treatment in the public healthcare system, making a statement that “my experience is a wake-up call for us in Kenya to build comprehensive cancer care centres for our own people, and for the East Africa region. I know we can do it if we begin by walking the talk” [26].

Both ministers publicly declared a renewed commitment to the fight against cancer and NCDs in general. This was the platform on which a Cancer Control Strategy was formulated and a Cancer Control Act legislated by government, both in the process of implementation.

#### 3.2.3. Cultural factors: The question of ‘research culture” and its relationship with aid dependence and global policy influence

As recounted by respondents, there has continuously been little political will to fund research within the country. Research in Kenya is primarily funded and undertaken by donor and academic institutions and individual students.

To blame for this status, as conveyed by informants, is the lack of a “research culture” amongst policy makers and government officials. A system whereby decision and policy making is either completely devoid of evidence basis, or is informed by unreliable/unrepresentative data has existed unchallenged for a long time, favoured by the wholesome adoption of global policy directives in response to international pressure and as conditions set for donor funding.

The first comprehensive policy document on NCDs by the government, the National Non Communicable Diseases Strategic Plan 2012–2017 [27], is in the process of being formulated by the MoH, development partners, civil and professional groups, and is due for launching in 2014. Conspicuously, the document does not base its strategies and proposed activities on local evidence. It however, primarily notes the lack of representative data and sets as its first agenda the establishment of baseline information on NCD burden and risk factor patterns.

This problem is not unique to LMICs such as Kenya, with a study of high income countries reporting figures as low as 28% of policy makers in Australia referring to research in decision making, with comparable findings elsewhere [28]. Reasons given for this discrepancy in research production and its use ranged from similar non-evidence based decision making culture to competing influences in policy making.

This repetitive and deeply concerning theme of lack of a “research culture” encountered in the study as a major impediment to prioritization of NCDs and by extension the establishment of NCD surveillance structures can be explained by both endogenous and exogenous factor.

##### Endogenous Factors

Lack of the technical know-how to analyze, interpret and therefore apply information from research was a running theme used to explain the lack of a research culture in Kenya. A study carried out in four low income countries (Malawi, Tanzania, Pakistan and India) reported similar lack of research culture attributed to the lack of research interpretation know-how amongst policy-makers, who would therefore not fully understand how to interpret and apply available information [29].

Other factors include competing interests, which more likely than not influence policy making. These factors may be *ad hoc* emergent issues requiring attention (such as the unprecedented HIV emergence), or political factors.

##### Exogenous factors

External factors contributing to the non-use of evidence based material in informing decision making can be explained by two interlinked concepts, both directly attributable to the international community's involvement in the country's policies. In the first instance, foreign aid being the primary driver of research locally and the detriments of that system will be discussed. Secondly, the country's system of policy making through which international directives govern local policy making with little reference to context-specific evidence.

The finding of international focus as a major factor in the punctuation of health policy with regards to NCDs in the Kenyan case is not surprising as the country has a strong history of policy-making highly reflective of global tidal waves which is exacerbated by the reliance on foreign bilateral and multilateral aid for the funding of policy implementation. Donor funds contributed to 35% of the total health expenditure in Kenya in 2009/10, according to the NHA [19]. Furthermore, research in Kenya is almost entirely funded by donors. Therefore, the government loses ownership of the process, with the research topics and process being dictated by the financier.

Studies of aid dependency in Sub Saharan Africa show that States which derive large proportions of revenue from international communities are less accountable to the electorate and are under less pressure to maintain popular legitimacy, consequently having less incentive to invest in public institutions, the aid hence doing more harm than good in the long run [30].

While foreign aid in Kenya has had a positive effect in causes such as HIV control, polio eradication and improvement of maternal and child health, detrimental effects of aid dependence have also been experienced. Budgets highly supplemented by donor funds are unreliable, as demonstrated by the global economic crisis, with major donor agencies cutting back aid as demonstrated by the Global Fund to Fight AIDS, Tuberculosis and Malaria announcing in 2011 that it would make no new grants until 2014 [31]. Donor funding is similarly unsustainable, and countries have to device internal income generating and distribution mechanisms which have inbuilt longevity.

Donor-funded research has been reported as unused and unusable in the developing world contexts described by the study on evidence-based policy making cited above, citing shortcomings such as the inability of external research organizations to identify priorities and understand cultural and contextual issues surrounding the research, rendering results irrelevant due to disengagement of policy makers [29]. Similarly, this lack of internal involvement leads to barriers in disseminating findings to policy-makers for use due to their disengagement in the research process.

A shift away from aid-dependent research will prove a beneficial move for the country, as that will pave way for internally funded and sustainable research mechanisms and systems. Research initiated and funded internally is more likely to reflect the public health priorities of the country as the choice of focus areas will be endogenous to the area of which benefits will be accrued. Another benefit of a shift away from aid-funded research is the increased relevance of the research findings in informing policy making and the ownership of the process will instigate the application of the findings in policy making as internally generated findings present greater motivation for their subsequent utilization. This will eliminate the third-party stance of government, effectively creating a “research culture”. This change will also create the need for accountability and efficiency, which will be achieved through monitoring, a process which in essence demands the establishment of a surveillance program.

The second aspect of foreign aid's detriment to the research culture in Kenya is the country's unquestioning and wholesome adoption of policies set by global institutions in response to pressure, and as conditionalities for aid set by development partners, rather than reliance on evidence-based context-tested self-generated policies. This system nullifies the need for derivation of local data for planning, therefore serving as an obvious detriment to the establishment of disease and risk factor surveillance programs.

An example of the implications of this blind shadowing of international trends has been described in a paper explicating the policy environment surrounding family planning in Kenya, recounting the commitment displayed by the government to family planning in the 1980s, in keeping with international political trends and pressure [32]. However, in the 1990s, weakened prioritization of reproductive health in international policy agendas undermined local access to contraceptive services; this despite evidence to the sustained high fertility rates. This quagmire was only dealt with through active and intensive advocacy from strong civil society groups within the country.

Departure from wholesome adoption of international prescriptions into local policy in favour of homegrown evidence-based policy making will automatically inspire motivation to produce national data on which to base these policies, and therefore, would serve as a key driver of the development of an NCD risk factor surveillance program.

A symbiotic relationship between the government and international agencies should be fostered to replace the current opportunistic interaction; with internal initiative guiding policy making with international community offering a second-order driving force. In such an environment, nationally representative data will prove imperative for progress, motivating the NCD surveillance plan.

### 3.3. Process

The policy process has been defined as “the way in which policies are initiated, developed or formulated, negotiated, communicated, implemented and evaluated” [12].

The theoretical model of Baumgartner and Jones, namely, the Punctuated Equilibrium theorem explains the policy making as occurring in transient bursts proceeding and following periods of relative inertia [33]. In health policy, this is characterized by long periods when a disease entity or public health issue's priority remains relatively unchanging, with interventions confined to select populations followed by bursts of priority and focus, typically attributable to significant internal or external events [34]. This theory is based on a mechanistic self-correcting cycle concept of negative and positive feedback processes, representing the periods of unchanging status quo and punctuations of significant systemic changes respectively, reinforcing each other to create equilibrium.

While long periods of intensive effort were applied by local advocates in an endeavour to widen the policy stance, according to the findings, the major shifts made in policy and activities surrounding NCDs followed transient endogenous and exogenous incidents, described as the “Situational Factors” above. Of these, the most significant changes have been in response to international focus on NCDs and to the public cancer battles of key political figures.

**Figure 2.**
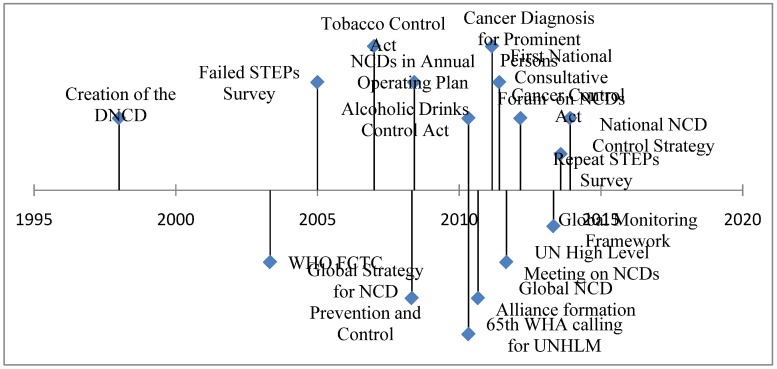
International and National NCD events and factors in Kenya since 1995.

The timeline above (Figure 2) illustrates this phenomenon according to the findings, demonstrating the relative quiescence in NCDs followed by the sudden flurry of activity coinciding with the most significant situational events.

With this knowledge in mind, the process is hereby described using the Sabatier and Jenkins-Smith “stages heuristic” approach which uses a four step system as outlined below [35]. It is worth noting that a limitation experienced by most researchers, including this one, in using this theoretical framework is the complexity of most policy environments coupled with the heterogeneity of involved actors, making it difficult to describe the stages in the linear model the framework presents. Also worth mention is that with no specific policy on NCD risk factor surveillance in existence, this section aims to detail the processes currently in motion with regards to the subject at hand, in an attempt to detect the bottleneck at which surveillance in Kenya has been impeded. In this vein, the “evaluation” stage has been utilized to detail the failures experienced thus far in relation to the topic at hand.

#### 3.3.1. Problem identification

As pointed out by most of the stakeholders interviewed, one of the greatest challenges to the creation of a comprehensive NCD policy, beyond the political contextual factors discussed above, is the lack of supporting evidence in the form of nationally representative data on the magnitude of the NCD problem in Kenya.

As one informant stated “while attempting to identify problem areas, we looked at the HMIS, we were surprised to find that NCDs are not even properly captured in the data system and the reporting system in the Ministry. This was our problem, if they are not well captured, how do you measure the burden of the problem you're trying to sell?”

An unambiguous acknowledgment of the necessity of a baseline NCD risk factor survey was recounted. The importance of the exposition of the current NCD disease burden and its risk factor pattern was deemed crucial for evidence-based prevention and control strategy formulation.

A focus on risk factors was advanced as particularly important, and was urged as a starting point in creating the NCD picture in Kenya, as risk factor pattern information was considered “easiest to act on immediately with the most significant results”. Of particular interest was risk factor data in the young, as this was assumed the best entry point for intervention strategies.

Repetition of the surveys and integration of NCD risk factors in the HMIS, forming a viable surveillance system was also identified as a critical undertaking. The arguments reported for the formulation of a surveillance strategy included the importance of surveillance in tracking progress made by interventions, and therefore reorganization and re-strategization accordingly.

#### 3.3.2. Policy formulation

Following the identification of the need for risk factor data, and in the heels of the failed 2005 STEPS survey, a repeat survey is underway for implementation tentatively within 2014. The process is led by the MoH, with prime collaborations with WHO, CDC and KEMRI, and wide consultation of stakeholders within private sector, professional associations and civil society.

The survey is receiving financial support from the CDC. The WHO involvement includes the provision of technical support, along with technical equipment (the personal digital assistant (PDA) data collection instruments), training of data collecting personnel and sensitization of communities through direct supervision of the pilot. KEMRI, beyond technical and implementation support, is also mandated with the responsibility of guidance on matters of ethics, as the third step involving biochemical testing which requires invasive procedures presents an ethical quandary.

#### 3.3.3. Implementation

##### 2014 Survey implementation

The upcoming STEPS survey is a household-based national survey, with sampling being carried out by the Kenya National Bureau of Statistics. The sample population will include everyone aged 15 years and above, a change from the recommended 17 years and above, introduced to capture the alcohol consumption, smoking and unprotected sex aspects, which have been previously identified in various studies as initiating in a younger age group in Kenya.

The STEPwise approach includes a section with optional questions of which countries can choose to include or not include in their surveys. Kenya has included injuries as that is an aspect of NCDs of critical importance.

##### Long-term surveillance implementation

While a consensus was found on the need for a long term risk factor surveillance strategy, a clear outline is yet to be formulated, with most quarters citing the success and results of the impending STEPs survey as a foundation for the designing the long term system. This section will therefore present the variety of options suggested by stakeholders on a surveillance program formulation.

A multiplicity of opinions on the design of a risk factor surveillance component was encountered due to the fact that facility data cannot reliably be representative of population level exposure. One school of thought proposed the setting up of sentinel sites in high-risk zones, as informed by the findings of the upcoming survey. Periodic surveys would then be carried out in these areas to track the trend of exposure and the efficacy of interventions installed.

A second proposition suggested integration of risk factor indicators such as smoking, alcohol intake and anthropometric measures into the existing national household-based Kenya Demographics and Health Survey, carried out 5 yearly by the Kenya National Bureau of Statistics nationwide. Yet another school of thought is the periodic repetition an independent national STEPS survey on a 5-yearly basis.

#### 3.3.4. Evaluation

The 2014 STEPS survey is yet to be implemented and therefore cannot be evaluated. Concerns that came up prior to implementation ranged from technical capacity, funding to quality.

The STEPS survey is set to be funded by the CDC, which is reportedly funding a parallel national survey on tobacco use, namely Global Adult Tobacco Survey (GATS). Concern over the MoH capacity to run two large-scale surveys of such magnitude simultaneously has been cited. Competition for resources is feared to cause failure of one or both surveys, or the production of questionable quality of data.

The question of the MoH's capacity to carry out large scale studies, GATS notwithstanding, also came up as a repetitive concern, especially in light of past failures. Other quarters differed with this proposition, displaying faith in the present strength of the NCD agenda and its consequent effect on the heightened commitment of the government in the success of NCD related activities such as the survey.

An important point worth noting is the lack of any records or information on the failed 2005 STEPS survey. The different sources cited different opinions on the reasons for the failure, reiterating the lack of information on the same. This implies that no evaluation post-survey was carried out to ascertain the problem areas for the benefit of future attempts.

However, the findings generally pointed to the factors below as the causative elements in the failure of the previous survey:

##### Minimal stakeholder involvement

Most of the interviewed stakeholders claimed lack of consultation and involvement in the survey process. Resources at the disposal of the various offices including technical assistance, training and intellectual input in the design process, were not exploited as afforded. The Ministry seemed, as one informant puts it, “to want to go it alone”.

##### Methodological errors

Unclear sampling procedure was indicated as making the resultant data unusable, due its unreliability, inability to be weighted and inability to establish its representatively. In the process of designing the study, multiple subtle alterations were reportedly made to the questionnaire collectively changing the nature of the indicator being tested, therefore making the process unstandardized. An example of such an alteration was the change of the question “Do you smoke daily?” to “Do you smoke regularly?”, two questions whose answers imply completely different aspects of the indicator in question. Also, advancements in data collection tools from hand-written records to hand-held electronic devices less capable of error occurred in 2006, a year after the Kenyan survey attempt, during which data was collected in the archaic manual fashion. Accurate and detailed accounts of the survey process were also not readily available, bringing into question the scientific rigor applied, consequently resulting in dubious findings.

### 3.4. Content

This section outlines the policy provisions and recommendations of the various NCD related policy documents and directives, both at national and international level, with regards to risk factor surveillance.

#### 3.4.1. Global policies and directives

The issue of risk factor surveillance has been at the heart of the NCD discussion on a global level for a significant period of time. The illustrious 2011 UN High Level Meeting (UNHLM) culminated with attending Heads of State signing a resolution document whose declarations included a pledge by member states “…….to increase and prioritize budgetary allocation for addressing non-communicable disease risk factors and for surveillance, prevention, early detection…….” [36].

Prior to the UNHLM, the Action Plan for Global Strategy for the Prevention and Control of Non-communicable Diseases, 2008–2013 [37] which was launched by WHO at the conclusion of the 61st World Health Assembly and adopted by all signatory member states for national level implementation, had one of its six main objectives as “To monitor non-communicable diseases and their determinants and evaluate progress at the national, regional and global levels”, proposing the establishment of high-quality surveillance and monitoring systems to provide population-based statistics on mortality, key NCD risk factors and behavioral patterns based on WHO STEPwise approach.

As part of the resolution of the sixty-sixth World Health Assembly in May 2013, a Global Monitoring Framework which outlined nine targets and 25 indicators was duly adopted by member states [25]. It was developed following the UNHLM 2011 political declaration on NCDs to enable global monitoring and tracking of progress in preventing and controlling the major NCDs and their key risk factors as a foundation for advocacy, political commitments and promotion of global action.

## 4. Discussion

The two major documents analyzed are both drafts of yet to be released policy papers, namely the Health Sector Strategic Plan III [21] and the National Non Communicable Diseases Strategic Plan 2012–2017 [27].

Three of the proposed six objectives in the HSSP III relate to NCDs, aiming to a) halt and reverse the rising burden of NCDs, b) reduce the burden of violence and injuries, and c) minimize exposure to health risk factors. The third mentioned objective, however, serves the whole spectrum of diseases and is not restricted to NCDs.

The strategy places emphasis on the strengthening of the health information system to provide adequate health information for evidence-based decision making. It goes on to outline the weaknesses of the existing health information system (DHIS2), the health system limitations including technology, infrastructure and human resources which limit DHIS2s efficacy, finally proposing various methods of improving it.

The document also emphasizes the importance of surveys and surveillance systems which should be developed and fostered to provide population data on disease and risk factor trends. Although the document does not explicitly address the issue of NCD risk factor surveillance, it does demonstrate intent by policy makers on prioritizing surveillance.

The National Non Communicable Diseases Strategic Plan 2012–2017 has been modelled on the 61st WHAs Global Strategy for NCDs 2008–2013 [37], containing ten strategies, the first six of which have been adapted from the GAP, and an additional four strategies outlining objectives for injuries, environmental and occupational hazards, capacity building and healthcare system strengthening.

Strategy 1 proposes the undertaking of a national situation analysis for NCD burden and risk factors through STEPS survey and patient outcome indicator data collection within the 2012–3 period by the MoH and DNCD. Strategy 4, dedicated to the promotion of research for the prevention and control of NCDs, outlines the conduction of baseline surveys and periodic surveillance of NCDs and their risk factors as a research objective.

Strategy 6 is dedicated to surveillance, reading “To promote monitoring and surveillance of non-communicable diseases and their determinants and evaluate progress at the national and county levels”, going on to explain the importance of the establishment of effective monitoring and evaluation systems in continuously measuring the progress and impact of the implementation of NCD policies in assuring achievement of planned interventions; impact evaluation for assessment of collective efforts by stakeholders; and effective and sustainable program implementation.

The strategy proposes the development of NCD M&E indicators and integrating them into the DHIS, dissemination of the same and establishment of a database to strengthen collection, reporting, analysis and utilization of NCD data at all levels of the healthcare system. It also proposes periodic review of NCD plans being implemented.

## 5. Conclusion

Progress has positively been achieved in Kenya in the recognition of NCDs. The upcoming STEPS survey is a good starting point for the formation of a long-term NCD risk factor surveillance strategy. However, the hurdles likely to be encountered in moving forward, as elucidated above, include the lack of a “research culture”, fostered by international policy and aid dependency, and manifested in policy making devoid of context-specific evidence-based interventions. This external reliance has played a role in the absence of baseline NCD risk factor and epidemiologic data, with a failed STEPS survey in 2005 due for repetition in 2014. Dissociation from this dependency is encouraged, in favour of a symbiotic health diplomatic angle, from which Kenya's policies and funding primarily derive internally, with collaboration with international initiatives serving to provide second-order technical advice and guidance.

Areas of opportunity include the highly active, visible and vocal civil and professional societies, unified into the Kenya National NCD Alliance. The exploitation of this resource for technical leverage both nationally and globally for sensitization and promotion of the NCD agenda would prove highly beneficial, as this network extends into a global umbrella system.

Finally, it is imperative for the stakeholders to get back to the drawing table and outline a strategic approach to NCD risk factor surveillance in Kenya. Recommendations to undertake a large-scale situational analysis prior to the establishment of an NCD risk factor surveillance system have been made, and this research can be considered to set the stage for one.
